# Commentary on “Visualization of Intraoperative Pancreatic Leakage (Vip): The IDEAL Stage I First-In Human, Single-Arm Clinical Pilot Trial of Smartpan”

**DOI:** 10.1097/AS9.0000000000000544

**Published:** 2025-03-11

**Authors:** Martin de Santibanes

**Affiliations:** From the Department of General Surgery, Hepato-Pancreato-Biliary and Liver Transplant Unit, Hospital Italiano de Buenos Aires, Argentina, Buenos Aires, Argentina.

The development and initial testing of SmartPAN presents a promising innovation in the field of pancreatic surgery, particularly in managing the complications associated with partial pancreatectomy, such as postoperative pancreatic fistula (POPF).^1^ POPF, which affects up to 50% of patients following these surgeries, is a high-risk complication that can lead to significant morbidity, longer hospital stays, and even mortality.^2–4^ The primary benefit of SmartPAN is its ability to detect pancreatic enzyme leaks in real time by changing color when in contact with alkaline fluids typical of pancreatic excretions. This intraoperative feedback could allow surgeons to address potential leak sites immediately, potentially reducing the risk of POPF. Given the high rates of POPF and associated healthcare burdens, this tool’s utility is clear in identifying and mitigating leaks that might otherwise be undetectable intraoperatively.

SmartPAN’s simplicity and usability, demonstrated by surgeon-reported ease of use and observed effectiveness, suggest that it could be a valuable adjunct to standard pancreatic surgery protocols. The immediate color change in response to leaks enables precise localization of potential leakage points, allowing for targeted repairs during surgery. In this trial, surgeons frequently used SmartPAN’s feedback to apply extra sutures where leaks were detected, often achieving successful closures. However, some surgeons noted limitations in the durability and precision of the color change, which may indicate a need for further refinement of the indicator’s visual response to ensure it remains clear and reliable throughout the procedure.

In terms of safety, the trial indicates that SmartPAN has a favorable profile, with no adverse events directly attributable to its use. Importantly, no detectable bromothymol blue – the active component – was found in patients’ blood or drainage fluids postoperatively, highlighting its low systemic impact and compatibility with safe surgical practice.^5,6^ Although POPF still occurred in some patients despite SmartPAN’s application, the fact that clinically relevant leaks were lower among cases where the indicator was used suggests potential benefits that could be further examined in larger, randomized trials.

For broader clinical application, SmartPAN could become a standard component of pancreatic surgeries in high-risk patients, especially where partial resections are required, and POPF risk is elevated. Its use could extend beyond the scope of this initial trial if further studies confirm its effectiveness in reducing postoperative complications and hospital stays. Furthermore, adapting SmartPAN for laparoscopic and robotic surgeries could enhance its versatility in minimally invasive procedures, aligning with the trend toward less invasive approaches in pancreatic surgery.
